# Differences in Antenatal Care Policies in England, Finland, and the Netherlands: A Framing analysis

**DOI:** 10.1007/s10995-023-03882-3

**Published:** 2024-02-09

**Authors:** Hanna Wierenga, Mats Målqvist

**Affiliations:** Department of Women’s and Children’s Health, Uppsala University, Uppsala, Sweden

**Keywords:** Antenatal care, Policies, Framing analysis, Country comparison

## Abstract

**Background and Purpose:**

The World Health Organization (WHO) states that good quality antenatal care should strive for both mother and child achieving their best possible health. On a policy level, in Europe these goals are reached with varying approaches. This research offers a fresh look on the underlying assumptions embedded in the ANC policies in three European countries.

**Methods:**

A framing analysis was conducted to publicly available ANC policies on uncomplicated pregnancies in Finland, England, and the Netherlands. Analysis was guided by van Hulst and Yanowa and included the following phases: a) Sense-making, b) Selecting, naming, and categorizing and c) Storytelling.

**Main Findings:**

Findings of this study demonstrate how ANC is organized with distinct frames. The Finnish ANC policies emphasized equity in care and instead of focusing on women, the ANC focused on the family. In England the pregnant woman was central, and it is seen as her responsibility to understand the ANC protocols. The ANC in the Netherlands focused on the pregnant woman’s pregnancy experience and freedom.

**Conclusion:**

The three studied countries had individual priorities and values guiding ANC provision. Despite each country being in line with the WHO ANC recommendations, areas requiring improvement should not be overlooked.

**Supplementary Information:**

The online version contains supplementary material available at 10.1007/s10995-023-03882-3.

## Significance

**What is Already Known on this Subject?:**

Antenatal care is organized differently in European countries depending on their health system. These varying approaches lead to women’s unequal access to good quality care during their pregnancies. Despite maternal mortality being the lowest in Europe compared to other continents, disparities and challenges remain.

**What this Study Adds?:**

This study contributes with improved understanding on how antenatal care aims to achieve good maternal health in three European countries by framing the ANC in Finland, England, and the Netherlands.

**Supplementary Information:**

The online version contains supplementary material available at 10.1007/s10995-023-03882-3.

## Introduction

According to the World Health Organization (WHO), the aim of antenatal care (ANC) is to reach the best possible health for women and child during pregnancy (World Health Organization, 2016). This happens through frequent visits to the ANC, meeting the midwife and/or obstetrician, taking certain blood samples, measuring blood pressure or discussing topics essential to the pregnancy period (Geller et al., 2018; World Health Organization, 2016). Nevertheless, there are context specific factors shaping the provision of ANC (Tenbensel et al., 2012; Zeitlin et al., 2019).

In Europe, differences in the organization of care arise from multiple dimensions leading to varying pregnancy outcomes (World Health Organization, 2023; Zeitlin et al., 2019). Funding, the political agenda, or health system related issues are all factors that shape the ANC (Tenbensel et al., 2012). Additionally, the timely interest in public health initiatives and sociodemographic characteristics of the population can influence the extension and quality of health promotion for pregnant women (Tenbensel et al., 2012; Zeitlin et al., 2019). In this paper with the word “women” is referred to the sex at birth. However, while acknowledging that this categorizing might be misleading, it is used due to convenience, and reflecting the previous literature (Magliozzi et al., 2016).

Fetal health goes hand in hand with the woman’s health during pregnancy, and the ultrasound scan (US) is a powerful tool for monitoring this (Kaelin Agten et al., 2021). It is possible to follow the growth of the fetus, detect abnormal development, or it can serve as a way to connect with the unborn child, depending on the pregnant woman’s wishes (Ekelin et al., 2004; Kaelin Agten et al., 2021).There is no clear consensus on when the US should happen and in the country’s policy on timing of the US affects the detection of congenital anomalies during pregnancy (Curado & Bhide, 2018).

Additionally, pregnant women are a diverse group, and the ANC should adapt to their needs when conducting health promotion. Especially as the mean age of women giving birth is getting on average higher and the BMI of pregnant women is increasing (Mills et al., 2011; Nohr et al., 2009). Hence, ANC policies should adapt to the timely needs of the pregnant women in order to continuously provide good quality care (Koblinsky et al., 2016).

Discrepancies between guidelines emphasizes the importance of evidence-based policies. However, to generate evidence based policies in pregnancy care, the current ANC policies should be better understood (Daviter, 2015). With a policy analysis disparities in the political agenda can be better understood (Parsons, 2002). Hence, the aim of this study is to investigate the underlying assumptions or values presented in the ANC and ultra sound (US) policies, by analyzing publicly available documents in England, Finland, and the Netherlands (Browne et al., 2019; Tenbensel et al., 2012).

### Theoretical Framework

The social cognitive theory states that an individual’s self-efficacy is at the core of the health promotion (Bandura, 2004). This is also shown in women’s health and thus it is used in this study. Empowerment on sexual and reproductive health and rights leads to improved pregnancy outcomes (Upadhyay et al., 2014). The social cognitive theory recognizes that to achieve health, it is required to not only include biomedical means. This approach is also closely relevant to the ANC, as achieving the full potential of pregnancy does not solely mean the absence of diseases or survival (Lattof et al., 2020; World Health Organization, 2016). To conduct successful health promotion on an individual level, it is essential to look at the health system more broadly. ANC can be seen as an opportunity to improve a woman’s health also beyond the pregnancy and reaching a positive public health impact (Langer et al., 2015).

## Methodology

ANC and US policy documents from three European countries: England, Finland, and the Netherlands were collected and qualitatively analyzed. The chosen countries have both similarities and differences in the organization of their ANC.

### Context

In England the health care is publicly funded and provided by the National Health Service (NHS) (*NHS* *England*, 2023). The ANC is midwife-led, but lack of workforce poses challenged (Boyle, 2011). Similarly, in Finland ANC is funded through taxes, free at the point of care and led by a specialized nurse. The ANC is planned and regulated on a national level but the ANC is provided by the municipally (Tasa et al., 2021). In the Netherlands a hybrid insurance-based health care system has a minimum mandatory health insurance and private clinics (Kroneman & van den Berg, 2016). The basic ANC services are included in the mandatory health insurance and women can choose their ANC from the available providers.

### Data Collection

In March 2022, data was collected in the native languages (ENG, FI, NL). The search words “maternity care”, “antenatal care”, “pregnancy ultrasound” and “fetal screening” were used to search webpages of the policy or decision-making bodies regulating ANC. Most recent documents and webpages targeting the pregnant woman or the professionals working directly with the provision of the ANC were included. In the Netherlands, there are two tracks a pregnant woman can follow when entering ANC. If she has a risk of complications, e.g., during multiple births pregnancy or an advanced age, she will have ANC in the tertiary hospital. If the pregnancy is expected to progress without complications, she can choose the care provider herself. The policy texts collected for this study concerned the latter case.

Documents/webpages were excluded if they had updated versions or were predominantly on complications, multiple birth pregnancies, infections during pregnancies, birth defects, fetal development. Documents and/or webpages were identified until no more relevant information was found. Nothing needed to be translated as one of the authors (HW) is fluent in all three languages. The full list of the analyzed material is in the appendix. While conducting this research, the COREQ guidelines for qualitative research have been followed (O’Brien et al., 2014; Tong et al., 2007).

### Data Analysis

This qualitative study used the ‘framing analysis’ that has been developed to analyze and understand policy texts (Koon et al., 2016). It is important to distance framing (analysis) from the words *frame or framework*, often a structured concept setting boundaries or defining the perspective (Koon et al., 2016). Framing analysis on the other hand, is an inductive method aiming to give insight to the assumptions and underlying values communicated through the text in the policies (Entman, 1993; van Hulst & Yanow, 2016). Scheufele and Iyengar metaphorically describe that a painting with a richly decorated frame can look different if displayed in a simple frame (Scheufele & Iyengar, 2014). This is the same for messages communicated in the policy text. The content of the policy might be similar between countries, but it can be represented with emphasis on different aspects (Koon et al., 2016). Hence, framing analysis is a tool that can be used to identify these differences between policy texts (Koon et al., 2016; van Hulst & Yanow, 2016).

**Fig. 1:**
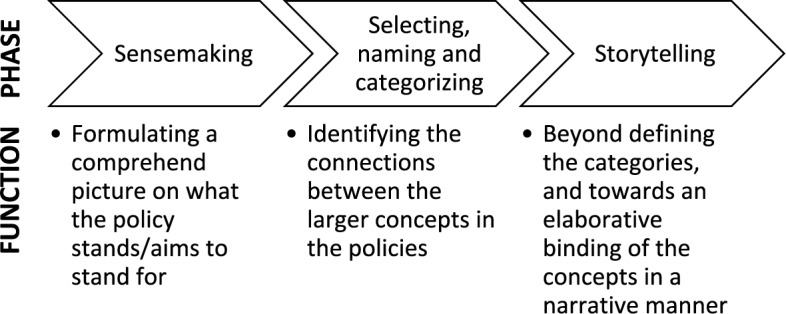
The framing analysis process used for this data analysis as presented by van Hulst and Yanow 2016

We performed the framing analysis as presented by van Hulst and Yanow while taking into account the recommendations on framing analysis by Koon et al. (2016) (Fig. 1). In the first phase (sensemaking), the data was read and uploaded to NVIVO 1.6.1 for the first round of inductive coding one country at a time. Those pages relevant for the research were coded line-by-line by the first author (HW) in their original language. After each country the authors reflected and discussed the codes to improve the quality of data extraction.

In the second phase (selecting, naming, and categorizing), the codes were double checked, by going through each document a second time and recoding them. Extracted data was placed into a concept map to identify the categories. Especially in this phase, a notebook was used to reflect on the overarching message in the policy texts. From this phase there are quotes in the results section.

We entered the third phase of the study (storytelling) by discussing and reflecting upon the codes, and concept maps. Before writing the results down, the codes and concept maps were looked through once more to make sure nothing was left out. Each country was handled separately to get a clear understanding of the frames in each country.

## Results

Each studied country had their own frame in which the ANC was presented. This means differences in the wording of ANC policies and guidelines, leading to differences in approaches of the ANC services. This was most prominent regarding the focus of care, and making decisions about the ANC trajectory. In England, the pregnant woman is the focus, and she is provided with information and makes the decision about care. In Finland, on the other hand, the health authority’s main focus is the wellbeing of the unborn child. The ANC guides the decisions about the pregnancy care based on what is seen as best for the family as a unit. In the Netherlands, the health care provider’s main concern is to make sure that major complications are avoided. Within this frame, the pregnant woman and her stress-free pregnancy experience is the focus.

The findings also showed that there were differences in the amount of openly available information regarding the ANC policies and guidelines between countries, with England having the least and Finland the most (Table 1).

**Table 1:** Publicly available documents on antenatal care and ultrasounds practices. (Reference to the used document in Appendix)

Country	Amount of ultrasound scan related documents (pages)	Amount of antenatal care related documents (pages)	Number of documents concerning both ultrasound scan and antenatal care	Total amount of pages	Total amount of documents
England	7 (33)	5 (78)	0	111	12
Finland	2 (214)	4 (128)	1 (412)	547	7
The Netherlands	3 (134)	3 (109)	1 (59)	243	7

### England—Listen And Respond

When caring for a pregnant woman, listen to her and be responsive to her needs and preferences*.*

(National Institute for Health and Care Excellence. Antenatal care)

In England the ANC should prevent complications and aim to encourage women to be the leaders of their pregnancy. It is the ANC specialist’s responsibility to equip the pregnant women with the necessary information about the pregnancy process so that she can make the decision about her care. Additionally, the ANC workers can only communicate with other professionals (for example for referral) if permission is given by the pregnant woman.

At your booking appointment, your midwife will put your details in a record book and add to them at each appointment. These are your maternity notes, sometimes called handheld notes. You’ll take your maternity notes home and be asked to bring them to all your antenatal appointments. Take your notes with you wherever you go in case you need medical attention while you’re away from home.

(National Health Service. Your antenatal care)

In the first visit, women are asked about their life situation. After this, she is encouraged to mention to the ANC worker if her situation changes. This might include violence from the partner or having economic challenges. Additionally, health promotion is emphasized on the first visit and not systematically covered in the following ANC visit.

You’ll be weighed at your booking appointment, but you will not be weighed regularly during your pregnancy*.*

(National Health Service. Antenatal check and tests)

The pregnant woman decides whether she participates in the screening based on the information given during ANC visits. If she decides to participate in the US screening, her understanding of what is screened for will be tested at the point of care by the sonographer. This will happen through written consent, and if the understanding is not sufficient, more information will be shared before trying to reach the consent again.

Ensure that when offering any assessment, intervention or procedure, the risks, benefits and implications are discussed with the woman and she is aware that she has a right to decline. … Women’s decisions should be respected.

(National Institute for Health and Care Excellence. Antenatal care)

### Finland—Active Involvement in the Family Unit

In Finland, the health authority is responsible for protecting and looking after families and children. The Finnish evidence-based ANC system is protected and directed by the law. Despite the fetus not having any legal rights, it is seen as the society’s task to protect the unborn child. There is a standardized structure for organizing ANC visits including a nation-wide protocol for the ANC visits. All pregnant women are expected to use the public ANC services, and if visits are skipped the public ANC evaluates the need for additional support.

Risks related to pregnancy and childbirth, or to fetal and child health, or risks of injury, must be prevented throughout the pregnancy. Although the child is a member of the family and community into which he or she is born, it is the responsibility of health professionals to assess the family’s individual needs for help and support. The newborn child is vulnerable and needs special protection*.*

(Klementti R, Hakulinen-Viitanen T. Antenatal care manual)

The ANC in Finland has a holistic approach to health promotion. The ANC aims to create a bond with the pregnant woman to get a comprehensive overview of the life situation she and the co-parent are in. Continuously in the ANC visits, the health and wellbeing of the pregnant women will be discussed. Ideally, the health promotion in the ANC has a positive long-lasting effect on health, while also reaching the co-parent. However, if challenges occur, for example in mental health, economic stability or the social environment, the pregnant woman and/or the family will be offered additional support. The policies also acknowledge that being pregnant and getting a child are life-changing events and additional support should be offered with a low threshold.

From the perspective of the individual and the family, health promotion is about empowering the individual and the family and, where necessary, achieving change. Health promotion emphasizes awareness, motivation, activity, care, responsibility and coping, and the possibility of adaptation*.*

(Klementti R, Hakulinen-Viitanen T. Antenatal care manual)

The ANC services have three assumptions that are frequently present in the ANC policies. Firstly, when a pregnant woman enters the ANC, she allows her information to be shared with other health care professionals, which can only be prohibited upon requests. The collaboration with other professionals and health facilities is emphasized. Secondly, it is assumed that the pregnant woman is living with the co-parent and this co-parent is male. Throughout the ANC visits, becoming or being a parent is discussed as well as how a child might influence the relationship of the parents and vice versa. Thirdly, it is assumed that the pregnant woman visits the US screenings both in the first and second trimester to conduct any abnormality screenings.

From the parents' point of view, the aim of the maternity clinic is that parents know what changes pregnancy, childbirth and upbringing bring of the child or childcare will bring to their personal and family life, and have the tools to handle them. The parents feel that they have been empowered to grow as mothers and fathers and to manage their relationship*.*

(Klementti R, Hakulinen-Viitanen T. Antenatal care manual)

### The Netherlands—Good Enough Care Brings Freedom

In the Netherlands, individualized care is one of the guiding principles. The ANC in the Netherlands is emphasizing women’s freedom to choose what kind of ANC or delivery care she gets. Pregnancy is seen as a unique process that will cause a change in the woman’s body and can awaken both positive and negative emotions. It is also mentioned that this might happen to a possible co-parent. There is no structured nationwide health promotion plan, as it is something that the pregnant woman and the ANC worker discuss if needed.

How are you feeling? Are you happy to be pregnant? Or the opposite?

(National Institute for Public Health and the Environment. Ministry of Health, Welfare and Sport. Pregnant!)

The screening is presented in the policy texts as something that will be done to an unborn child and the pregnant woman needs to be informed and prepared for any possible findings. Thus there are several steps before the US screening is completed.

The ultrasound result may reassure you. But the result may also worry you. Or scare you. Therefore, it is important that you think carefully whether you want the tests for physical abnormalities*.*

(National Institute for Public Health and the Environment. Ministry of Health, Welfare and Sport. Pregnant!)

The aim is to avoid over-medicalization but offer good enough care to pregnant women so that possible complications are detected and treated. Thus, there are two structural factors that shape the ANC in the Netherlands. Firstly, the collaboration between care providers is seen as one of the cornerstones to good ANC. However, as there is no digital solution for this, every pregnant woman gets a case manager from the ANC services. They have a central role in the communication between professionals in ANC and the pregnant women and are seen as necessary for good care.

The Care Standard was drawn up from the perspective of the pregnant woman and is intended to be the national standard with which integrated birth care must comply in terms of content, organization, and quality. The care standard is thus a 'living' document with room for innovation, allowing research findings to be incorporated in subsequent version

(Sluijter A. Pregnancy and delivery)

The second structural factor encourages women to think about their delivery beforehand. This is both, to prepare for delivery and because of administrative reasons. Women need a referral to a hospital or a birth center if they prefer not to give birth at home. Hospital delivery is free of charge if the referral is made by a general practitioner, or in case of an emergency. Women should be equipped with enough knowledge and understanding of the different options to be able to prepare for the delivery.

Together with your midwife or obstetrician, you make a birth plan. In a birth plan, you can write down what you think is important during childbirth and after your baby is born. Together with your midwife or obstetrician, you will decide whether your wishes are feasible*.*

(National Institute for Public Health and the Environment. Ministry of Health, Welfare and Sport. Pregnant!)

## Discussion

Each country, England, Finland, and the Netherlands had framed their ANC guidelines with a different approach. Despite all being high-income countries, with a health care system that strives for universal health coverage, the ANC policies had differences regarding the focus of care and how women´s health was supported during the pregnancy period. Socio-cultural norms and the health system specific reasons might lead to the ANC in England and the Netherlands focusing on empowering women during pregnancy and Finland framing the pregnant woman as a member of the family as a unit (Tenbensel et al., 2012; Zeitlin et al., 2019). On the other, women’s autonomy meant in England that she had the responsibility of a successful ANC, while in the Netherlands and Finland the ANC accommodated anything that hinders a successful ANC.

From a health system perspective, in the case of the Netherlands, with an insurance-based health system, pregnant women might expect higher level of influence in the decisions around care and thus it is a more marketized environment (Reibling & Zagel, 2021). In 2010 findings in the PERISTAT study showed the maternal mortality and morbidity outcomes were lagging behind compared to other European countries (De Jonge et al., 2013). This started an ANC reform that has made ANC policies for some parts more structured while still allowing women to personalized their pregnancy care (Vos et al., 2016). In Finland and England, the ANC is dominated by public providers. In England the ANC is focused on preventing pregnancy complications while in Finland the ANC is seen as an opportunity for health promotion.

The sociodemographic differences also influence what kind of care women get at the ANC (Tenbensel et al., 2012). In England large socioeconomic inequalities lead to health inequalities among pregnant woman and newborns and thus for the public health it is beneficial to focus on prevention and empower women to take care of their health (Wilding et al., 2019). In Finland, on the other hand there is a need for health promotion as nearly one in five of the women experience gestational diabetes and one in 10 smokes during pregnancy (Paulo et al., 2021; Smedberg et al., 2014).

When looking at the WHO ANC guidelines *Recommendations on antenatal care for a positive pregnancy experience* 2016 the studied countries approach it with different perspectives (World Health Organization, 2016). The Netherlands for example focuses on the positive pregnancy experience, while Finland follows the recommendations regarding health promotion and England captures the woman´s decision making as an important corner stone. Furthermore, each country recognizes that ANC is a service that can support long term good health and bring public health benefits. However, future challenges, such as increasing mental ill health, obesity and hypertensive disorder should be focused on with more clarity. Thus, each country has their strength but should also keep learning from each other. Overall, women should get good quality ANC regardless of the geographical location (Koblinsky et al., 2016).

Regarding the methodological considerations, this analysis only included the written text from publicly available policies. The framing analysis could have been expanded to also include other forms of expression or historical aspects, such as political statements or the progression of the health system. Additionally, the focus was now on the ANC on a primary level, there might be more or different frames on the tertiary level. However, as this is the first of its kind, an improved understanding of ANC policies in the studied countries might benefit future policy makers.

## Conclusion

This study has investigated and found differences in the ANC and US policies in three separate countries in Europe. England having an emphasis on women’s decision making-autonomy, Finland emphasizing, the authority’s role in supporting the family as a unit while the Netherlands focuses on women´s pregnancy experience. The studied countries were partly in line with the WHO ANC recommendations. However, each country had different emphasize and to maintain a low maternal mortality and morbidity the studied countries could learn from each other for future policy making.

### Supplementary Information

Below is the link to the electronic supplementary material.

Supplementary file1 (DOCX 28 KB)
