# Supplementary material for: Differences in Antenatal Care Policies in England, Finland, and the Netherlands: A Framing analysis
Source: Matern Child Health J. 2024 Feb 9;28(4):738–45. doi: 10.1007/s10995-023-03882-3 (PMC10963453; doi:10.1007/s10995-023-03882-3)
Supplement: Supplementary file 1 — Supplementary file1 (DOCX 28 KB) [file 10995_2023_3882_MOESM1_ESM.docx]

Documents used for analysis and their references :

England

| **Name** | **Pages in document** | **Year** | **Provided by** |
| --- | --- | --- | --- |
| Antenatal care guideline (1) | 60 | 2021 | NICE Guideline |
| Antenatal checks and tests (2) | 3 | 2020 | NHS |
| Maternity transformation program (3) | 6 | 2020 | NHS |
| Your antenatal appointments (4) | 5 | 2019 | NHS |
| Your antenatal care (5) | 4 | 2017 | NHS |
| ANC total | 78 |  |  |
| 11 physical conditions (20-week scan) (6) | 5 | 2021 | Government publications |
| 12-week scan (7) | 3 | 2020 | NHS |
| 20-week screening scan (8) | 4 | 2021 | NHS |
| Fetal anomaly screening programme handbook (9) | 9 | 2021 | Government publications |
| Screening for Down’s syndrome, Edward’s syndrome and Patau’s syndrome (10) | 4 | 2021 | NHS |
| Screening in pregnancy: dating scan (11) | 4 | 2021 | Government publication |
| Ultrasound scans in pregnancy (12) | 4 | 2020 | NHS |
| US total | 33 |  |  |
| **ANC and US together** | **111** |  |  |

| **Name** | **Pages in document** | **Year** | **Provided by** |
| --- | --- | --- | --- |
| Meille tulee vauva (13)  *[We are having a baby]* | 99 | 2020 | THL |
| Neuko-tietokanta, äitiys- ja lastenneuvola (14)  *[Neuko-database maternity and child health clinic]* | 23 | 2021 and 2022 | THL |
| Sikiön kasvun ja voinnin seuranta neuvolassa (15)  *[Monitoring fetal growth and well-being in the antenatal care]* | 3 | 2020 | Duodecim, Terveyskirjasto |
| Äitiysneuvolaseuranta (16)  *[Antenatal care follow-up]* | 3 | 2021 | Duodecim, Terveyskirjasto |
| ANC total | 128 |  |  |
| Raskaudenaikainen sikiöseulonta (17)  *[Fetal monitoring during pregnancy]* | 3 | 2020 | Duodecim, Terveyskirjasto |
| Sikiötutkimukset (18)  *[Fetal examination]* | 4 | 2021 | Duodecim, Terveyskirjasto |
| US total | 7 |  |  |
| Äitiysneuvola opas (19)  *[Antenatal care manual]* | 412 | 2013 | THL |
| **ANC and US together** | **547** |  |  |

Finland

| **Name** | **Pages in document** | **Year** | **Provided by** |
| --- | --- | --- | --- |
| Basis prenatale zorg (20)  *[Basis antenatal care]* | 55 | 2015 | Federatie Medisch Specialisten |
| Zwangerschap en geboorte – Een gezonde start voor moeder en kind (21)  *[Pregnancy and birth – A healthy start for mother and child]* | 50 | 2016 | ZonMw |
| Verloskundig zorg voor de bevalling (22)  *[Obstetric care before the delivery]* | 4 | 2021 | RIVM |
| ANC total | 109 |  |  |
| De 13 wekenecho en de 20 weken echo (23)  *[The 13-week ultrasound scan and the 20-week ultrasound scan]* | 32 | 2022 | RIVM |
| De NIPT (24)  *[The NIPT]* | 36 | 2022 | RIVM |
| Draaiboek Prenatale Screening (25)  *[The antenatal care screening script]* | 66 | 2021 | RIVM |
| US total | 134 |  |  |
| Zwanger! (26)  *[Pregnant]* | 59 | 2021 | RIVM |
| **ANC and US together** | **243** |  |  |

The Netherlands

References to the data

ENGLAND

1. National Institute for Health and Care Excellence. Antenatal are [Internet]. 2021 Aug [cited 2022 May 5]. Available from: https://www.nice.org.uk/guidance/ng201/resources/antenatal-care-pdf-66143709695941
2. National Health Service. Antenatal checks and tests [Internet]. nhs.uk. 2020 [cited 2022 May 5]. Available from: https://www.nhs.uk/pregnancy/your-pregnancy-care/antenatal-checks-and-tests/
3. National Health Service. Maternity Transformation Programme [Internet]. [cited 2022 May 5]. Available from: https://www.england.nhs.uk/mat-transformation/
4. National Health Service. Your antenatal appointments [Internet]. nhs.uk. 2020 [cited 2022 May 5]. Available from: https://www.nhs.uk/pregnancy/your-pregnancy-care/your-antenatal-appointments/
5. National Health Service. Your antenatal care [Internet]. nhs.uk. 2020 [cited 2022 May 5]. Available from: https://www.nhs.uk/pregnancy/your-pregnancy-care/your-antenatal-care/
6. Public Health England. 11 physical conditions (20-week scan) [Internet]. GOV.UK. [cited 2022 May 5]. Available from: https://www.gov.uk/government/publications/screening-tests-for-you-and-your-baby/11-physical-conditions-20-week-scan
7. National Health Service. 12-week scan [Internet]. nhs.uk. 2020 [cited 2022 May 5]. Available from: https://www.nhs.uk/pregnancy/your-pregnancy-care/12-week-scan/
8. National Health Service. 20-week scan [Internet]. nhs.uk. 2020 [cited 2022 May 5]. Available from: https://www.nhs.uk/pregnancy/your-pregnancy-care/20-week-scan/
9. National Health Service. Fetal anomaly screening programme handbook [Internet]. GOV.UK. [cited 2022 May 5]. Available from: https://www.gov.uk/government/publications/fetal-anomaly-screening-programme-handbook/overview
10. National Health Service. Screening for Down’s syndrome, Edwards’ syndrome and Patau’s syndrome [Internet]. nhs.uk. 2020 [cited 2022 May 5]. Available from: https://www.nhs.uk/pregnancy/your-pregnancy-care/screening-for-downs-edwards-pataus-syndrome/
11. Public Health England. Screening in pregnancy: dating scan [Internet]. GOV.UK. [cited 2022 May 5]. Available from: https://www.gov.uk/government/publications/early-pregnancy-scan-brief-description/fetal-anomaly-screening-pregnancy-dating-scan
12. National Health Service. Ultrasound scans in pregnancy [Internet]. nhs.uk. 2020 [cited 2022 May 5]. Available from: https://www.nhs.uk/pregnancy/your-pregnancy-care/ultrasound-scans/

FINLAND

1. Terveyden ja hyvinvoinnin laitos. Meille tulee vauva. 2020;99.
2. Terveyden ja hyvinvoinnin laitos. NEUKO-tietokanta - Duodecim [Internet]. 2021 [cited 2022 May 5]. Available from: https://www.terveysportti.fi/apps/dtk/nko
3. Kustannus Oy Duodecim. Sikiön kasvun ja voinnin seuranta äitiysneuvolassa [Internet]. Duodecim Terveyskirjasto. [cited 2022 May 5]. Available from: https://www.terveyskirjasto.fi/odk00018
4. Tiitinen A. Äitiysneuvolaseuranta [Internet]. Duodecim Terveyskirjasto. 2022 [cited 2022 May 5]. Available from: https://www.terveyskirjasto.fi/dlk00186
5. Kustannus Oy Duodecim. Raskaudenaikainen sikiöseulonta [Internet]. Duodecim Terveyskirjasto. [cited 2022 May 5]. Available from: https://www.terveyskirjasto.fi/odk00017
6. Tiitinen A. Sikiötutkimukset [Internet]. Duodecim Terveyskirjasto. [cited 2022 May 5]. Available from: https://www.terveyskirjasto.fi/dlk00175
7. Klemetti R, Hakulinen-Viitanen T. Äitiysneuvolaopas - Suosituksia äitiysneuvolatoimintaan [Internet]. THL; 2013 [cited 2021 Dec 19]. Available from: https://www.julkari.fi/handle/10024/110521

THE NETHERLANDS

1. Nederlandse Vereniging voor Obstetrie en Gycologie. Basis prenatale zorg [Internet]. 2015 May [cited 2022 May 5]. Available from: https://richtlijnendatabase.nl/richtlijn/basis_prenatale_zorg/basis_prenatale_zorg_-_startpagina.html
2. Sluijter A. Zwangerschap en geboorte - Een gezonde start voor moeder en kind. 2016;50.
3. Ministerie van Volksgezondheid W en S. Verloskundige zorg (Zvw) - Verzekerde zorg - Zorginstituut Nederland [Internet]. Ministerie van Volksgezondheid, Welzijn en Sport; 2016 [cited 2022 May 5]. Available from: https://www.zorginstituutnederland.nl/Verzekerde+zorg/verloskundige-zorg-zvw
4. Rijksinstituut voor Volkgezondheid en Milieu, Ministerie van Volkgezondheid, Welzijn en Sport. De 13 wekenecho en de 20 wekenecho. 2022;32.
5. Rijksinstituut voor Volkgezondheid en Milieu, Ministerie van Volkgezondheid, Welzijn en Sport. De NIPT - Onderzoek naar down,- edwards- en patausyndroom [Internet]. 2022. Available from: https://www.pns.nl/sites/default/files/2022-04/Maart_2022_Folder_De_NIPT_4.pdf
6. Rijksinstituut voor Volkgezondheid en Milieu, Ministerie van Volkgezondheid, Welzijn en Sport. Draaiboek Prenatale Screening version 11.0. 2021;66.
7. Rijksinstituut voor Volkgezondheid en Milieu, Ministerie van Volkgezondheid, Welzijn en Sport. Zwanger! Landelijke folder van verloskundige, huisartsen, gynaecologen en kraamzorg [Internet]. 22. Available from: https://www.pns.nl/sites/default/files/2022-04/21407154_013502_Brochure%20Zwanger%21_TG__april2022%20%28def%29.pdf
